# Supplementary material for: Variable Molecular Weight Polymer Nanoparticles for Detection and Hyperthermia-Induced Chemotherapy of Colorectal Cancer
Source: Cancers (Basel). 2021 Sep 5;13(17):4472. doi: 10.3390/cancers13174472 (PMC8431470; doi:10.3390/cancers13174472)
Supplement: Supplementary file 1 [file cancers-13-04472-s001.zip › cancers-1315151-supplementary.pdf]

Supplementary Materials

			Mean (mV)	Area (%)	St Dev (mV)
Zeta Potential (mV):	-5.27	Peak 1:	-5.27	100.0	5.13
Zeta Deviation (mV):	5.13	Peak 2:	0.00	0.0	0.00
Conductivity (mS/cm):	0.914	Peak 3:	0.00	0.0	0.00

Result quality : Good

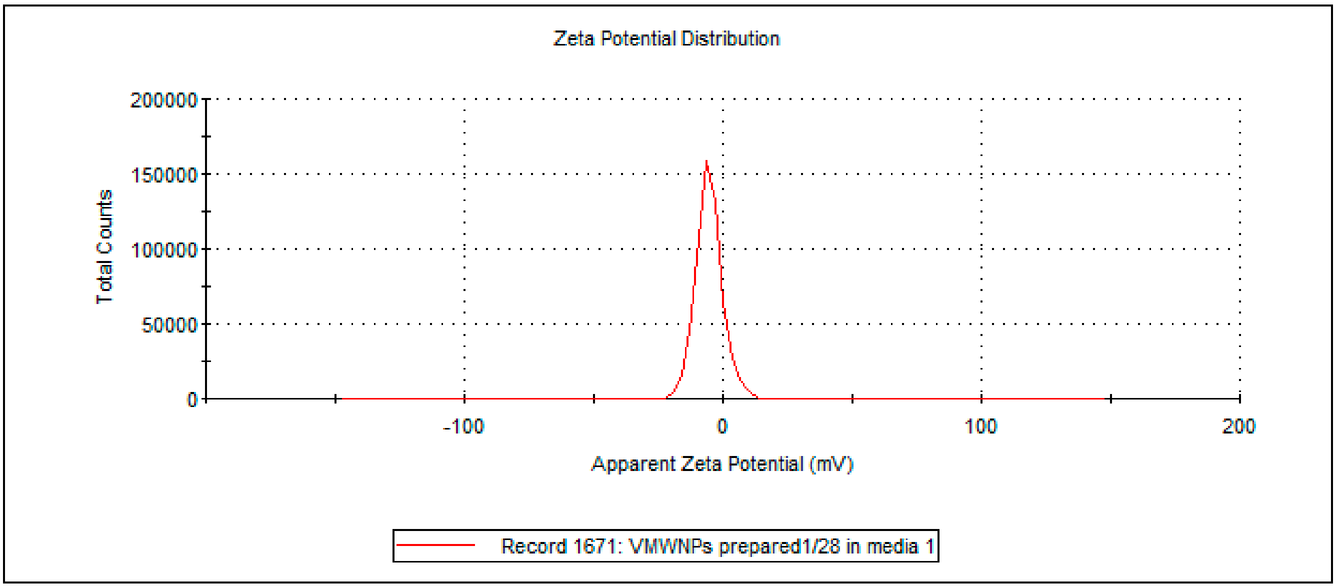

Figure S1. DLS analysis to evaluate zeta potential of VMWNPs.

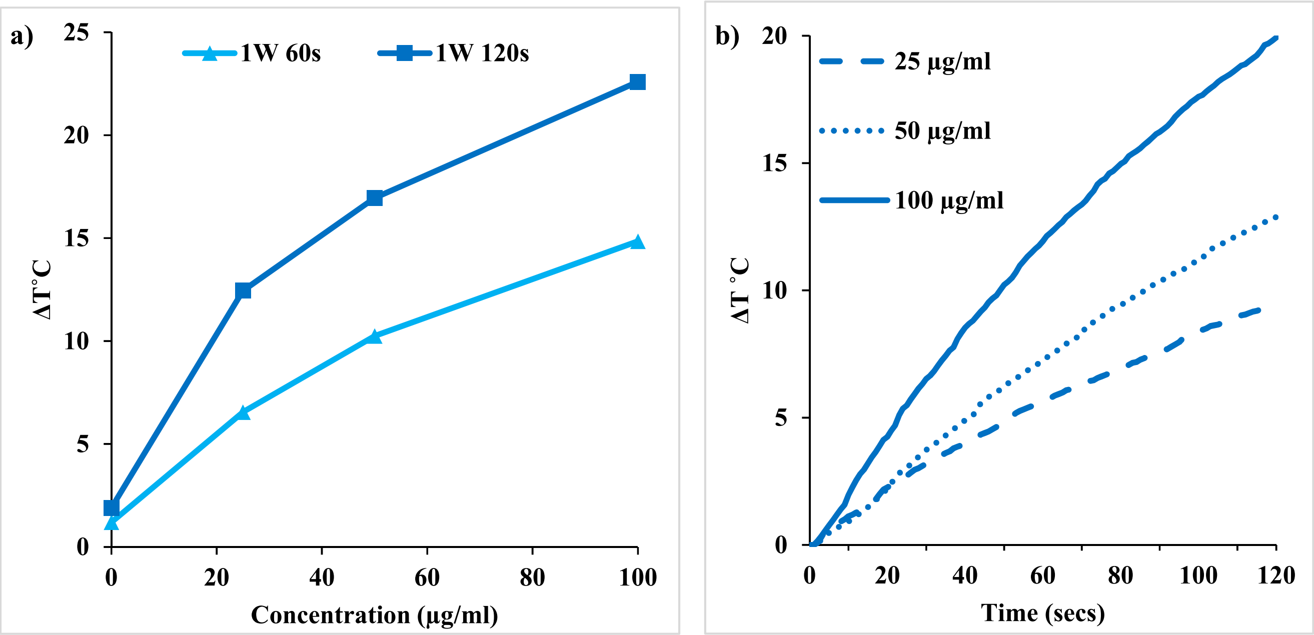

Figure S2. (**a**) Temperature variations of VMWNPs (300 µL) at different concentrations with 800 nm laser irradiation (1W 60 s & 1W 120 s). (**b**) Continuous measurement of temperature with 800 nm laser irradiations (1W 60 s) at different concentrations of VMWNPs.

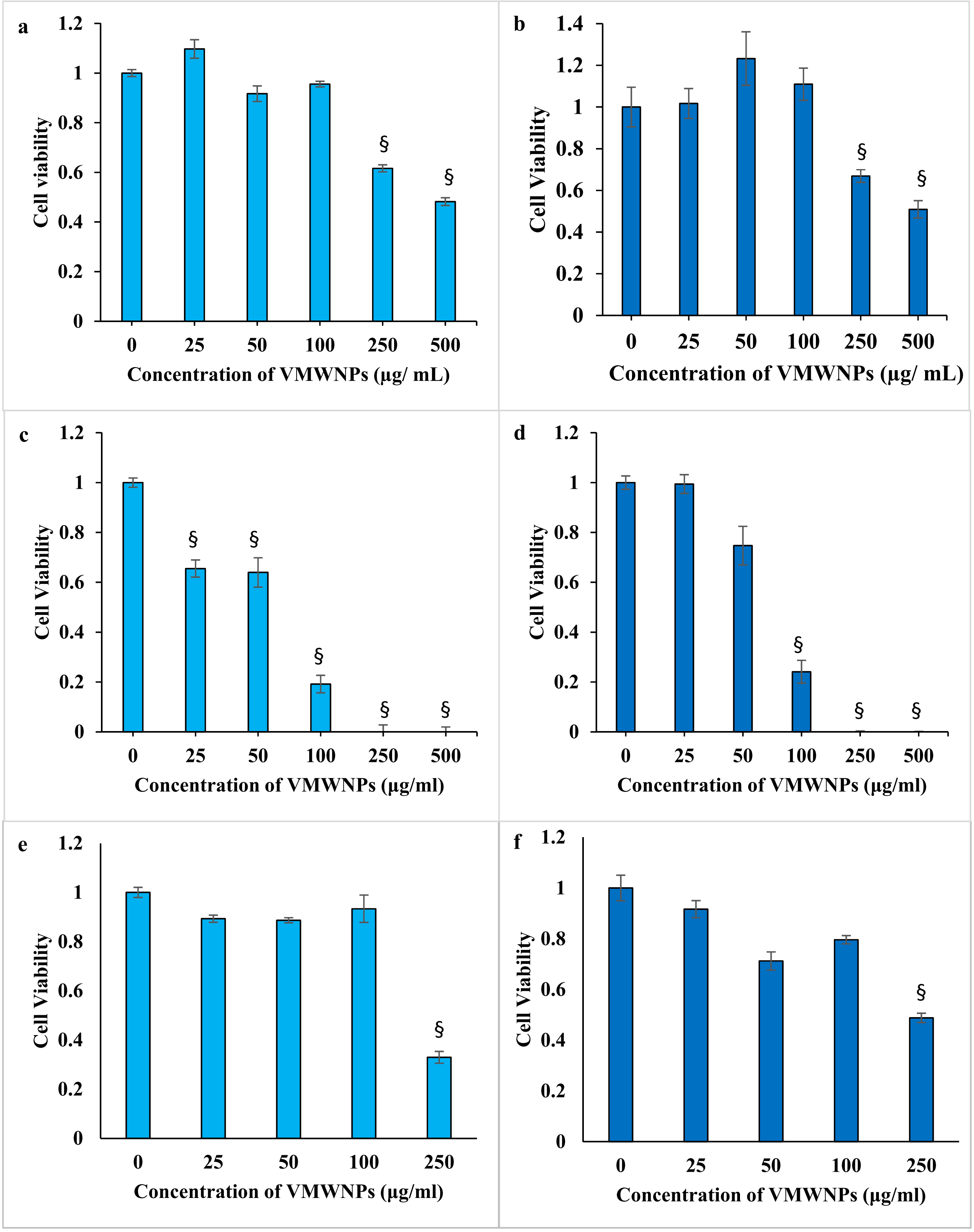

Figure S3. Cell viabilities of (**a**) OxS CT-26, (**b**) OxR CT-26, (**c**) OxS RKO, (**d**) OxR RKO, (**e**) OxS HT-29, and (**f**) OxR HT-29 CRC cells with increasing VMWNPs. § indicates statistical significance compared to the control (0 µg/ mL VMWNPs) ($p<0.05$).

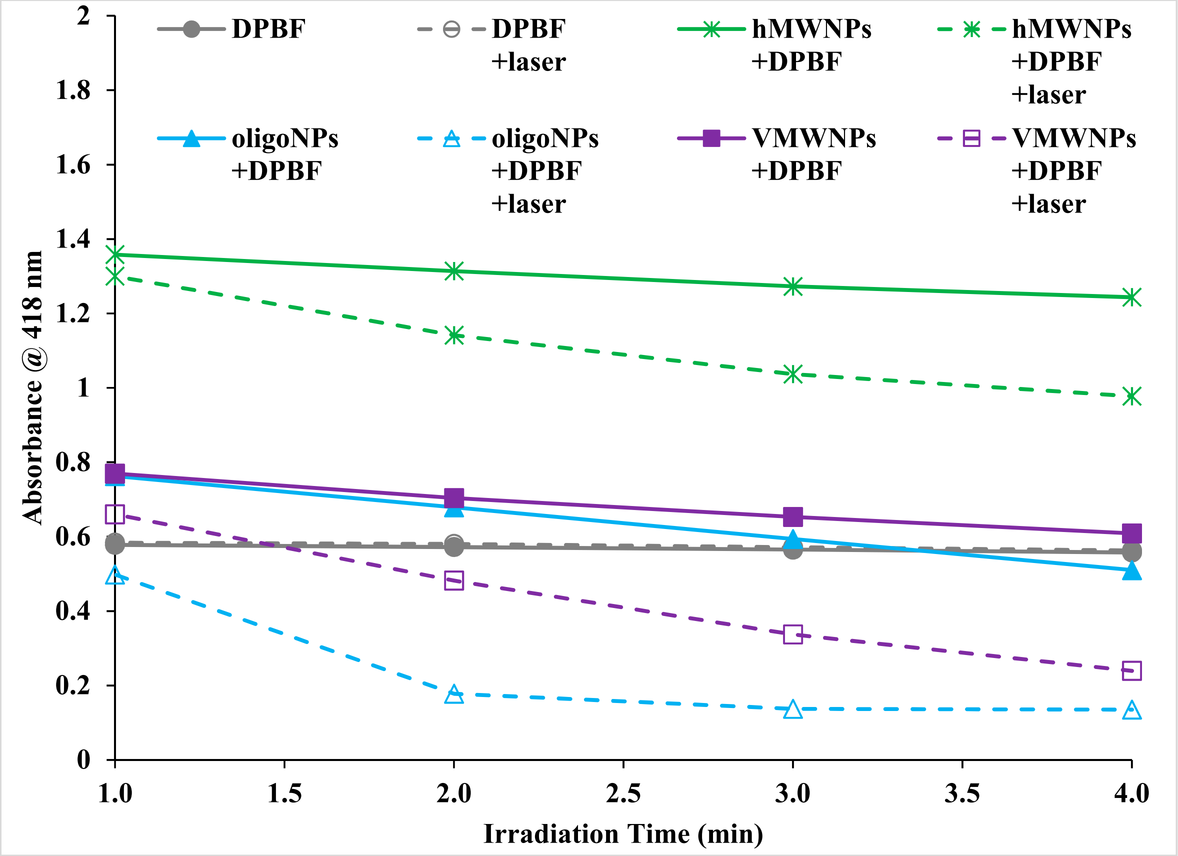

Figure S4. Absorbance of DPBF at 418 nm in the presence of hMWNPs, oligo NPs and VMWNPs with repetitive laser irradiation (1W, 60 s).

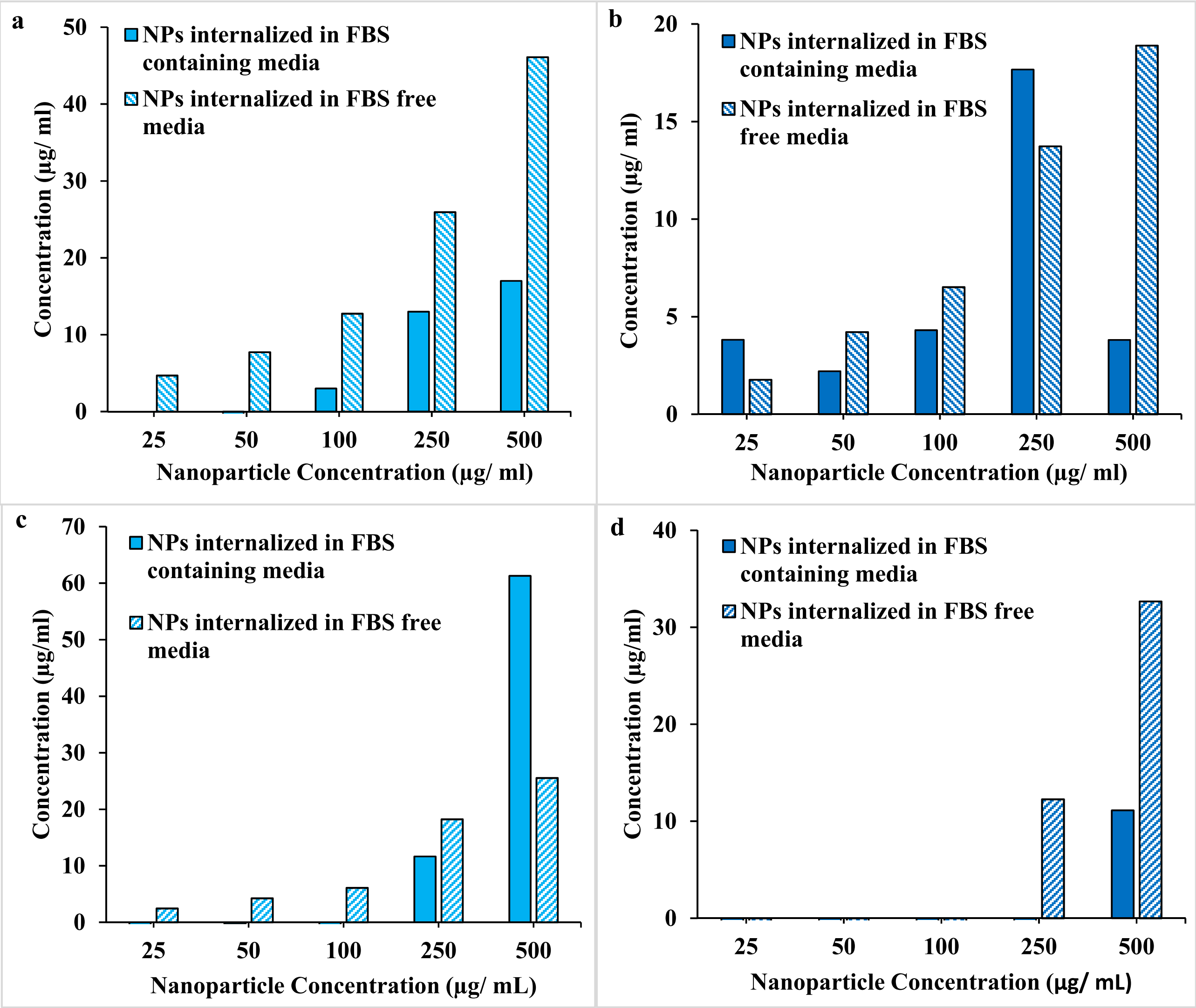

Figure S5. Intracellular concentrations of VMWNPs in FBS containing and FBS free media for (**a**) OxS CT-26, (**b**) OxR CT-26, (**c**) OxS RKO, and (**d**) OxR RKO CRC cells.

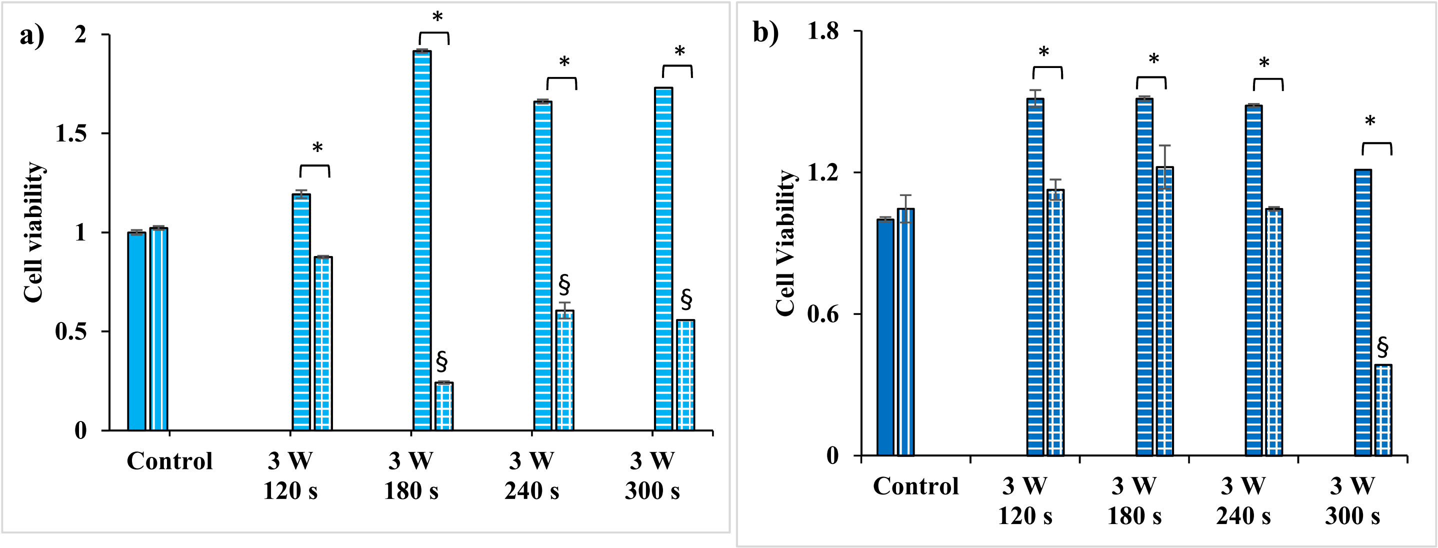

Figure S6. Cell viability of (**a**) OxS and (**b**) OxR CT-26 CRC cells following laser irradiation with and without internalization with VMWNPs. Solid bar represents cells treated with no VMWNP and no laser. Vertical stripes represent cells with internalized VMWNPs. Horizontal stripes represent laser stimulation and bars with both vertical and horizontal stripes represent laser application in cells with internalized VMWNPs. § indicates statistical significance compared to the control (0 µg/ mL VMWNPs and no laser stimulation) ($p<0.05$). * indicates statistical significance between groups ($p<0.05$).

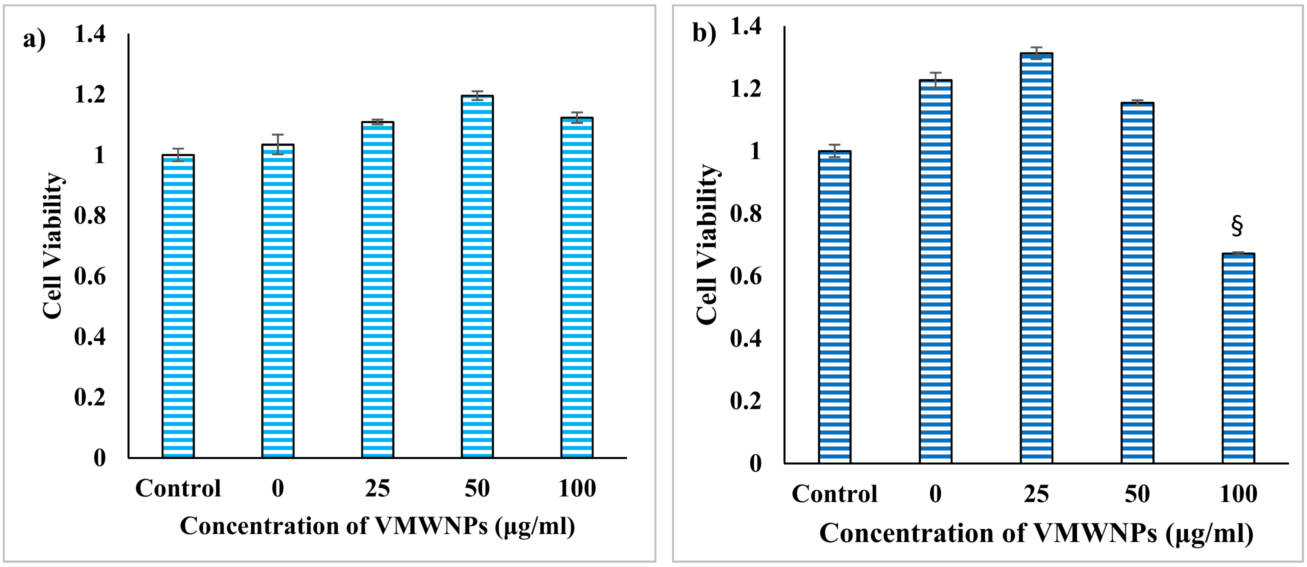

Figure S7. Cell viabilities of (**a**) OxS and (**b**) OxR HT-29 CRC cells with increasing extracellular concentrations of VMWNPs and 1W 60s of 800 nm light. § indicates statistical significance compared to the control (0 µg/ mL VMWNPs and no laser stimulation) ($p<0.05$).
